# T Cell Exhaustion in the Cervical Cancer Tumor Microenvironment: PD-1 Overexpression and Co-Expression with TIGIT, Tim-3, LAG-3, and NKG2A

**DOI:** 10.3390/cancers17223627

**Published:** 2025-11-11

**Authors:** Nadia Tatiana García-Barrientos, Fabiola Solorzano-Ibarra, Ksenia Klimov-Kravtchenko, Jose Manuel Rojas-Diaz, Marcela Sofia Guitron-Aviña, Francisco Javier Ceja-Flores, Jose Alfonso Cruz-Ramos, Pablo Cesar Ortiz-Lazareno, Felipe de Jesús Bustos-Rodriguez, Juan Carlos Vazquez-Limon, Miriam Ruth Bueno-Topete, Martha Cecilia Tellez-Bañuelos, Jesse Haramati, Susana del Toro-Arreola

**Affiliations:** 1 Instituto de Investigación en Enfermedades Crónico Degenerativas, Departamento de Biología Molecular y Genómica, Centro Universitario de Ciencias de la Salud, Universidad de Guadalajara, Colonia Independencia, Guadalajara 44340, Mexico; nadia.garcia2322@alumnos.udg.mx (N.T.G.-B.);; 2 Laboratorio de Inmunología Traslacional, Departamento de Biología Celular y Molecular, Centro Universitario de Ciencias Biológicas y Agropecuarias, Universidad de Guadalajara, Zapopan 45200, Mexico; 3 Coordinación de Investigación, Subdirección de Desarrollo Institucional, Instituto Jalisciense de Cancerología, Guadalajara 44200, Mexico; 4 Centro de Investigación Biomédica de Occidente, División de Inmunología, Instituto Mexicano del Seguro Social (IMSS), Guadalajara 44340, Mexico; 5 Departamento de Oncología del Hospital Civil de Guadalajara “Dr. Juan I. Menchaca”, Guadalajara 44340, Mexico; 6 Laboratorio de Inmunología, Departamento de Fisiología, Centro Universitario de Ciencias de la Salud, Universidad de Guadalajara, Guadalajara 44340, Mexico

**Keywords:** cervical cancer, checkpoint therapy, ICI, CIN, cervical dysplasia, chemoradiotherapy, TIGIT, LAG-3, PD-1, PD-L1

## Abstract

**Objective**: T cell exhaustion is a major mechanism of immune evasion in cancer, characterized by the sustained expression of multiple inhibitory receptors. This study aimed to evaluate the expression of immune checkpoints in peripheral and tumor-infiltrating CD8^+^ T cells from cervical cancer patients. **Methods**: We enrolled 104 participants: 37 treatment-naïve patients, 36 treated patients, and 31 age-matched healthy donors. Peripheral blood mononuclear cells (PBMCs) were isolated from all participants. Ten cervical biopsies were collected for tumor-infiltrating lymphocyte (TIL) isolation and paraffin fixation. Immune checkpoint expression was analyzed by multiparametric flow cytometry and immunohistochemistry. **Results**: In peripheral CD8^+^ T cells, we found a significant upregulation of exhaustion-associated markers PD-1, TIGIT, Tim-3, and LAG-3. In the tumor infiltrating lymphocytes, these same molecules, with the addition of NKG2A, were notably upregulated further. While BTLA and NKG2A showed no systemic changes, NKG2A increased in TILs and BTLA decreased in TILs. The co-expression of PD-1 with TIGIT, Tim-3, LAG-3, and NKG2A was notably enriched between 2- and 6-fold in TILs compared with patient PBMCs. The tumor microenvironment was highly immunosuppressive, characterized by enrichment with PD-1, PD-L1, and TIGIT; TIGIT was notably upregulated in locally advanced versus early-stage tumors. **Conclusions**: Our findings highlight the strongly immunosuppressive environment of cervical tumors in treatment-naïve patients and the presence of elevated inhibitory checkpoint expression in peripheral blood of both pre- and post-treatment patients. These results underscore the importance of investigating immune regulation within the tumor site itself and suggest that immune checkpoint co-expression may serve as a biomarker of T cell exhaustion and therapeutic resistance. Understanding how treatment alters these pathways could guide rational combination immunotherapies to restore CD8^+^ T cell function in cervical cancer.

## Simple Summary

Immune checkpoint inhibitor therapy is of growing importance for the treatment and management of cervical cancer. In this analysis of known and emerging inhibitory checkpoint receptors in patients with cervical cancer, we observed that in addition to an increase in PD-1 and TIGIT, the emerging receptors Tim-3, LAG-3 and NKG2A were likewise upregulated. The co-expression of multiple immune checkpoints indicates an interplay of exhaustion pathways and possible future therapeutic targets. The tumor microenvironment was highly immunosuppressive: T cells expressing multiple checkpoints were more likely to be found in the tumor than in peripheral blood mononuclear cells (PBMC)s, up to 6-fold higher in the case of PD-1+ NKG2A+ cells, highlighting the importance of examining biopsies, not only peripheral blood, for exhausted T cell signatures that may be key to cancer diagnosis and management. Understanding how treatment alters these pathways could support the development of rational combination immunotherapies to restore CD8^+^ T cell function in cervical cancer.

## 1. Introduction

Cervical cancer (CC) remains a major global health concern, with an estimated 369, 511 deaths reported in 2023 (range: 291,690 to 474,516 deaths) and 886,537 new cases reported in 2023 (range 682,464 to 1,173,087 cases) (GBD2023) [1]. Despite therapeutic advances, the five-year overall survival remains at 67%, and mortality is especially high in low- and middle-income countries due to limited access to prevention, early detection, and treatment. In Mexico, CC is the second most common cancer among women, with over 10,000 new cases in 2022, many at a locally advanced stage. This data underscores the urgent need to expand vaccination, improve screening, reduce health disparities, and advance personalized treatment strategies [2,3].

For early-stage cervical tumors, surgery and/or radiotherapy are the standard of care [4], while in locally advanced cases and metastatic disease, concurrent chemoradiotherapy, often platinum-based chemotherapy, remains the cornerstone [5]. Over the past decade, treatment strategies have expanded beyond conventional chemotherapy. The addition of bevacizumab, a VEGF inhibitor, to platinum-based regimens has improved outcomes in patients with recurrent, persistent, or metastatic CC who are ineligible for curative local therapies [6]. Most recently, immunotherapy, particularly approaches that enhance the cytotoxic function of CD8^+^ T cells, has emerged as a therapeutic option in CC management [7].

Despite these therapeutic advances, treatment resistance remains a major obstacle in cervical cancer management. Resistance to platinum-based chemotherapy and radiotherapy frequently leads to disease recurrence and poor survival outcomes, particularly in advanced stages. Mechanisms underlying this resistance include enhanced DNA repair capacity, altered apoptotic signaling, hypoxia-induced changes in the tumor microenvironment, and epithelial-to-mesenchymal transition [6]. Moreover, immune evasion contributes to therapeutic failure, as tumor cells can upregulate inhibitory ligands such as PD-L1, leading to T cell exhaustion and impaired cytotoxic responses [7]. Understanding how these mechanisms interact is essential for developing effective combination strategies that overcome resistance and improve the efficacy of immunotherapy in cervical cancer.

CD8^+^ T cells play a central role in antitumor immunity. Upon recognizing tumor-associated antigens, they release perforin and granzymes to induce apoptosis in cancer cells [8]. However, chronic antigen exposure can drive these cells into a dysfunctional state known as T cell exhaustion, marked by impaired proliferation, reduced cytotoxicity, and persistent expression of multiple inhibitory receptors [9]. Importantly, exhausted T cells retain the potential for functional recovery, positioning them as key targets for immunotherapeutic strategies [10].

Since the approval of the first immune checkpoint inhibitor, ipilimumab (anti-CTLA-4), immunotherapy has transformed cancer care [11]. Numerous studies have demonstrated the clinical efficacy of monoclonal antibodies that block immune checkpoint receptors, restoring CD8^+^ T cell function and enhancing antitumor immunity [12]. These agents are now integral to the standard of care for various cancers, used as monotherapy and in combination with other treatments [13]. To date, the FDA has approved seven checkpoint inhibitors targeting CTLA-4, PD-1, and PD-L1 [14].

The success of these therapies has triggered growing interest in additional inhibitory receptors implicated in T cell exhaustion. Molecules such as TIGIT, LAG-3, Tim-3, BTLA, and NKG2A have emerged as key regulators of dysfunctional T cells. However, their specific roles across tumor types, including cervical cancer, remain incompletely understood. Therefore, gaining insight into their expression patterns could reveal new opportunities for improving immunotherapeutic outcomes [15]. Therefore, this study aims to evaluate the expression of PD-1, TIGIT, LAG-3, Tim-3, BTLA, and NKG2A in CD8^+^ T cells from women with cervical cancer, both before and after treatment, in order to better understand their role in T cell dysfunction and identify potential targets for future therapeutic strategies.

## 2. Materials and Methods

### 2.1. Sample Collection

A total of 104 participants were enrolled in this study. Peripheral blood samples were obtained from patients with cervical cancer at the Antiguo Hospital Civil de Guadalajara “Fray Antonio Alcalde” and the OPD Instituto Jalisciense de Cancerología. The cohort was divided into three groups: 37 newly diagnosed, treatment-naïve patients; 36 patients receiving standard of care treatment, mainly systemic therapy (chemotherapy with or without radiotherapy), who had completed at least three treatment cycles; and an age-matched cohort, recruited over the course of the study, of healthy female donors with no history of malignancy (*n* = 31). In addition, tumor biopsies were collected from 10 treatment-naïve patients prior to therapy initiation. All participants provided written informed consent, and the study was conducted in compliance with institutional and national ethical guidelines.

Peripheral blood samples were collected in EDTA-coated tubes (Becton Dickinson, Franklin Lakes, NJ, USA, catalog: 366643), and peripheral blood mononuclear cells (PBMCs) were isolated by density gradient centrifugation using Lymphoprep (STEMCELL Technologies, Vancouver, BC, Canada; catalog: 07851). Tumor-infiltrating lymphocytes (TILs) were isolated from the tumor biopsies by enzymatic dissociation using enzyme A (Miltenyi Biotec, Bergisch Gladbach, Germany; catalog: 130-095-928) and enzyme H (Miltenyi Biotec, Bergisch Gladbach, Germany; catalog: 130-095-929). PBMCs and TILs were cryopreserved in a freezing medium composed of 90% fetal bovine serum (FBS) and 10% dimethyl sulfoxide (DMSO) and stored in liquid nitrogen until further use. Cell viability was assessed after thawing using trypan blue exclusion, and samples were interrogated via flow cytometric staining only when viability exceeded 90%.

### 2.2. Flow Cytometry

Immunophenotyping of the inhibitory receptors PD-1, TIGIT, LAG-3, Tim-3, BTLA, and NKG2A was performed on CD8^+^ T cells using flow cytometry. The following antibodies were used: CD3-FITC (Biolegend, San Diego, CA, USA; Clone: UCHT1; catalog: 300406), CD8-APC/Fire750 (Biolegend; Clone: SK1; catalog: 344746), PD-1-BV421 (Biolegend; Clone: EH12.2H7; catalog: 329920), TIGIT-PE (Biolegend; Clone: A15153G; catalog: 372704), LAG-3-PerCP/Cy5.5 (Biolegend; Clone: 11C3C65; catalog: 369312), Tim-3-BV5210 (Biolegend; Clone: F38-2E2; catalog: 345030), BTLA-PE/Cy7 (Biolegend; Clone: MIH26; catalog: 344516), and NKG2A-APC (Biolegend; Clone: S19004C; catalog: 375108). Additionally, for the analysis of TILs, we added CD45-AF700 (Biolegend; Clone: HI30; catalog: 304024).

Data acquisition was performed using the Attune NxT Acoustic Focusing Cytometer (Thermo Fisher Scientific, Waltham, MA, USA). Compensation was applied using compensation beads (BD Biosciences, Milpitas, CA, USA; catalog no. 55284). For each sample, 250,000 events were collected from the lymphocyte gate. Immunophenotypic analysis of peripheral and tumor-infiltrating T cells was performed using Kaluza software (version 2.1).

### 2.3. Immunohistochemistry

The biopsy samples, preserved in paraffin, from the treatment-naïve patients with a confirmed diagnosis of cervical cancer, were analyzed for the expression of immune checkpoints. Biopsies from nine of the ten patients detailed above were utilized for immunohistochemistry. The following antibodies were used: anti-PD-1 (Abcam, Cambridge, UK; Clone: EPR4877(2); catalog: ab137132), anti-TIGIT (Abcam; Clone: BLR047F; catalog: ab243903), anti-PD-L1 (Abcam; Clone: 73-10; catalog: ab228415), and anti-CD8α (Abcam; Clone: EPR21769; catalog: ab217344). Immunohistochemical staining was performed using the BOND-MAX fully automated IHC staining system (Leica Biosystems, Nussloch, Germany) according to the manufacturer’s recommended protocols. Human tonsil tissue was included as a positive control for all markers.

After immunohistochemical staining, high-resolution images were captured using a bright-field optical microscope at 10× magnification. Image analysis was performed with FIJI (ImageJ version 1.54 g, National Institutes of Health, USA; Java 1.8.0_345, 64-bit). To identify stained regions, we applied a semi-automated segmentation method using the “Color Threshold” tool, selecting areas based on hue and tone.

Criteria for thresholding: the threshold for image segmentation depends on the properties of the image, so a specific and unique threshold value was generated for each image. The default method used was based on an iterative procedure based on the IsoData algorithm, also called the iterative intermeans method [16]. This method segments images with iterations using the following formula: threshold = (mean background + mean objects)/2. An initial threshold is established, followed by the computation of the means of the pixel intensities equal to, below, or above that threshold. The mean of these two values is calculated, the threshold is increased, and the process is repeated until the threshold exceeds the composite mean.

In Fiji, using the multi-point tool, 100 points were selected by an expert pathologist for each high-power field (10 high-power fields were evaluated per case). Color thresholding was then performed (Image → Adjust → Color Threshold), automatic thresholds were removed, and the multipoints were selected as the threshold reference (Original → Sample). The color space setting used was HSB (Hue, Saturation, Brightness). The percentage of positive marking was calculated automatically per field, and the mean value of the 10 fields was calculated for each case studied; an average of 1000 to 3000 stained elements were quantified per image.

### 2.4. Statistical Analysis

Data normality was assessed using the D’Agostino–Pearson test. For datasets following a normal distribution, comparisons between two groups were performed using an unpaired *t*-test. Non-normally distributed data were analyzed using the Mann–Whitney U test. When comparing more than two groups, one-way ANOVA was applied, followed by appropriate post hoc analyses (e.g., Tukey’s multiple comparisons test) to identify specific differences between groups. *p*-values < 0.05 were considered statistically significant. All statistical analyses were carried out using GraphPad Prism v10.

## 3. Results

### 3.1. Clinical and Demographic Characteristics

A total of 104 participants were enrolled in this study and classified into three groups: newly diagnosed, treatment-naïve CC patients (*n* = 37); diagnosed patients with CC who were previously treated (*n* = 36); and age-matched healthy female donors with no history of malignancy (*n* = 31). Among CC patients, squamous cell carcinoma was the predominant histological subtype, observed in 76.7% of cases. Based on the FIGO staging system 2018, 80.8% were diagnosed at a locally advanced stage (IB2–IVA). Radiotherapy, either alone or combined with chemotherapy, was the primary treatment modality in 75% of patients. Clinical and demographic characteristics of all participants are detailed in Table 1.

### 3.2. Immune Checkpoint Expression Profile in Peripheral CD8^+^ T Cells

We first assessed the expression of major immune checkpoint receptors on peripheral CD8^+^ T cells in CC patients compared to HD (gating strategy is shown in Supplementary Figure S1). Among classical inhibitory receptors linked to T cell exhaustion, PD-1 expression was significantly increased in CC patients compared to HD (HD: 36.1%; CC: 45.1%). Similarly, TIGIT, a major suppressor of T cell activation and cytokine production, was upregulated in CC patients (HD: 37.3%; CC: 51.4%). Full results are detailed in Table 2.

**Figure 1:**
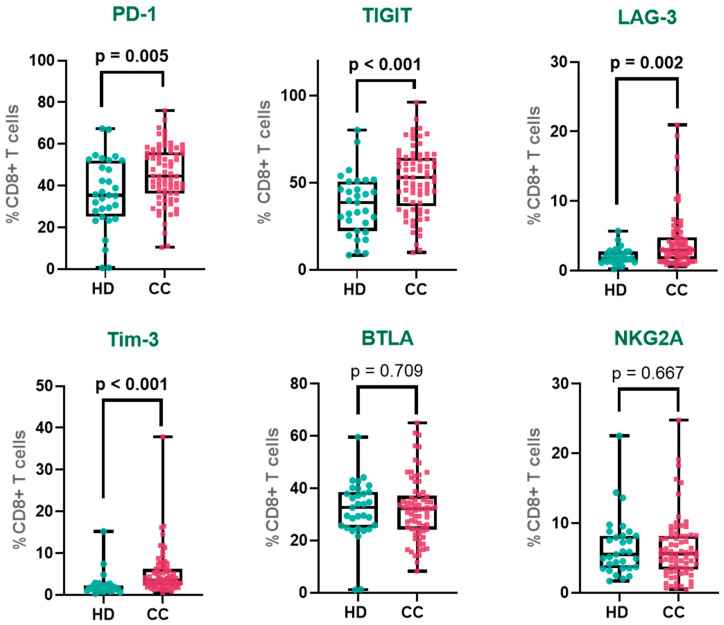
Immune checkpoint expression in peripheral CD8^+^ T cells. Percentages of CD8^+^ T cells expressing PD-1, TIGIT, LAG-3, Tim-3, BTLA, and NKG2A in cervical cancer patients and healthy donors. PD-1—Programmed Cell Death Protein 1, TIGIT—T cell Immunoreceptor with Ig and ITIM Domains, Tim-3—T cell Immunoglobulin and Mucin-domain Containing-3, LAG-3—Lymphocyte Activation Gene-3, NKG2A—Natural Killer Group 2 Member A and BTLA—B and T Lymphocyte Attenuator. The box represents the interquartile range (25th to 75th percentiles), with the center line indicating the median score. The whiskers extend to the min and max of the data set; bold numbers indicate significant results.

We also assessed a second tier of inhibitory receptors increasingly implicated in T cell dysfunction. LAG-3 expression was approximately two-fold higher in CC patients than in HD (HD: 2.1%; CC: 4.2%). Likewise, Tim-3, another inhibitory receptor associated with late-stage T cell exhaustion, was markedly elevated in CC patients (HD: 2.3%; CC: 5.2%). In contrast, BTLA and NKG2A, receptors involved in distinct immunoregulatory pathways, showed no significant differences between groups. BTLA expression was comparable in HD and CC patients (HD: 31.5%; CC: 32.1%), as was NKG2A (HD: 6.6%; CC: 6.3%) (Figure 1). Mean fluorescence intensity (MFI) was also evaluated; however, no significant differences were observed (Supplementary Figure S2). Additionally, we analyzed immune checkpoint receptor expression according to tumor histological subtypes, comparing squamous cell carcinoma (SCC) and adenocarcinoma (AC) samples. Our results showed that PD-1 expression was significantly higher in peripheral CD8^+^ T cells from patients with AC tumors compared to SCC, while the expression levels of other immune checkpoints did not differ between the subtypes (Supplementary Figure S3).

### 3.3. Differences in Immune Checkpoint Expression Between Treatment-Naïve and Treated Patients

To further investigate the impact of treatment on the expression of immune checkpoint receptors, we performed a one-way ANOVA to compare three groups: healthy donors (HD), treatment-naïve patients, and patients who had received treatment. This analysis allowed us to evaluate whether therapy modulated the expression of exhaustion-associated markers in peripheral CD8^+^ T cells (Table 2).

For PD-1, a gradual increase in expression was observed across the three groups, with a statistically significant difference detected between HD and treated patients (HD: 36.1%; treatment-naïve: 42.2%; treated: 48.2%). The expression in treated patients was approximately 1.3 times higher than in HD and also elevated compared to treatment-naïve patients, although this latter comparison did not reach statistical significance. In the case of TIGIT, expression was significantly increased in both cancer groups compared to HD (HD: 37.3%; treatment-naïve: 50.1%; treated: 52.8%). The difference between HD and treatment-naïve patients represented a 1.3-fold increase, while the expression in treated patients was approximately 1.4 times higher than in HD. However, no significant difference was detected between treatment-naïve and treated patients, suggesting stable expression following therapy.

For LAG-3, expression was lowest in HD and progressively increased in the patient groups (HD: 2.1%; treatment-naïve: 3.2%; treated: 5.2%). A statistically significant difference was found between treated patients and both HD and treatment-naïve patients. Expression in the treated group was approximately 2.5 times higher than in HD and 1.6 times higher than in treatment-naïve patients. Tim-3 expression followed a different pattern: while it was significantly higher in treatment-naïve patients compared to HD, corresponding to a 2.5-fold increase, in treated patients, expression decreased (HD: 2.3%; treatment-naïve: 5.7%; treated: 4.6%) and was no longer significantly different from HD, although still higher than baseline levels. This suggests that the increase observed in treatment-naïve patients was partially reduced following therapy.

**Figure 2:**
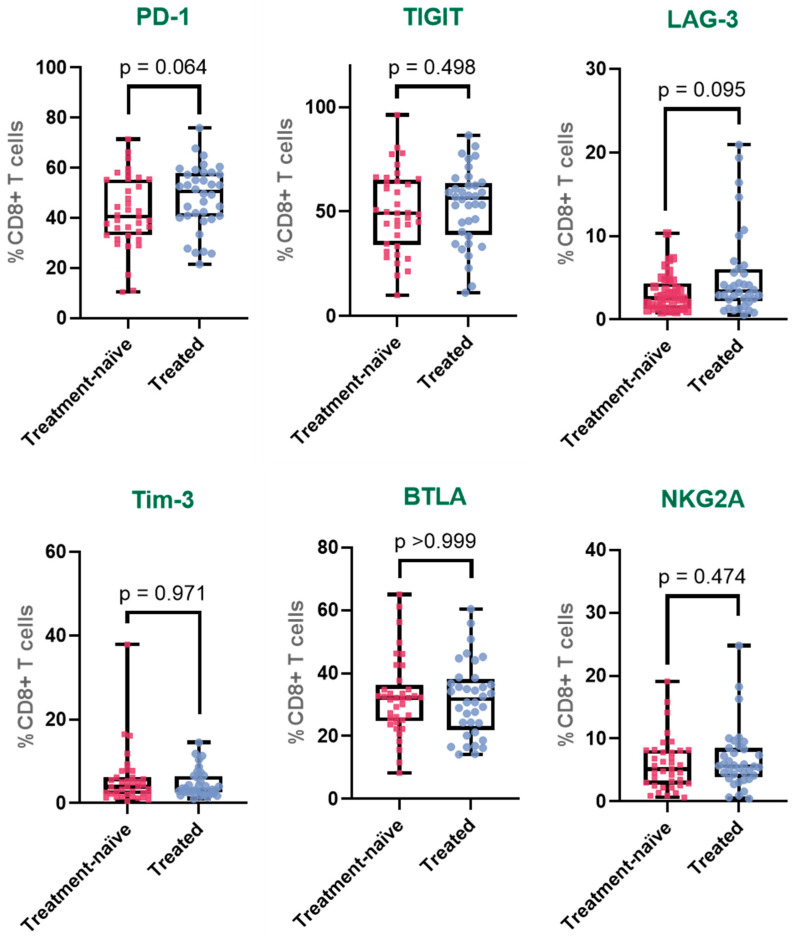
Immune checkpoint expression profiles in peripheral CD8^+^ T cells across clinical groups. Expression of PD-1, TIGIT, LAG-3, Tim-3, BTLA, and NKG2A on CD8^+^ T cells from healthy donors, treatment-naïve cervical cancer patients, and patients who received systemic treatment. The box represents the interquartile range (25th to 75th percentiles), with the center line indicating the median score. The whiskers extend to the min and max of the data set; bold numbers indicate significant results.

For BTLA, expression levels remained relatively stable across all groups (HD: 31.5%; treatment-naïve: 32.4%; treated: 31.7%). No statistically significant differences were observed between any pairwise comparisons. Similarly, NKG2A expression did not show significant variation across the groups (HD: 6.6%; treatment-naïve: 5.9%; treated: 6.7%). Expression remained consistent regardless of disease status or treatment exposure (Figure 2).

### 3.4. Immune Checkpoint Expression in Tumor-Infiltrating CD8^+^ T Cells

To investigate the immune inhibitory landscape of tumor-infiltrating CD8^+^ T cells, we analyzed the expression levels of multiple inhibitory receptors in CD8^+^ T cells isolated from the peripheral blood mononuclear cells (PBMCs) of 37 treatment-naïve cervical cancer patients and compared them to CD8^+^ T cells from tumor-infiltrating lymphocytes (TILs) obtained from 10 cervical tumor biopsies.

PD-1 was markedly upregulated in tumor-infiltrating CD8^+^ T cells, with expression levels nearly double those observed in peripheral CD8^+^ T cells (PBMCs: 42.2%; TILs: 79.0%), indicating a pronounced state of exhaustion within the tumor microenvironment. Similarly, TIGIT also showed elevated expression in the tumor-infiltrating CD8^+^ T cell population (PBMCs: 50.1%; TILs: 65.6%).

LAG-3 demonstrated a notable 2.7-fold increase in expression in TIL-derived CD8^+^ T cells compared to their peripheral counterparts (PBMCs: 3.2%; TILs: 8.6%), further supporting the accumulation of inhibitory receptor-expressing T cells within the tumor site. Similarly, Tim-3 was more than twice as prevalent in the tumor-infiltrating CD8^+^ T cell population (PBMCs: 5.7%; TILs: 13.7%), reflecting a phenotype commonly associated with terminal exhaustion.

**Figure 3:**
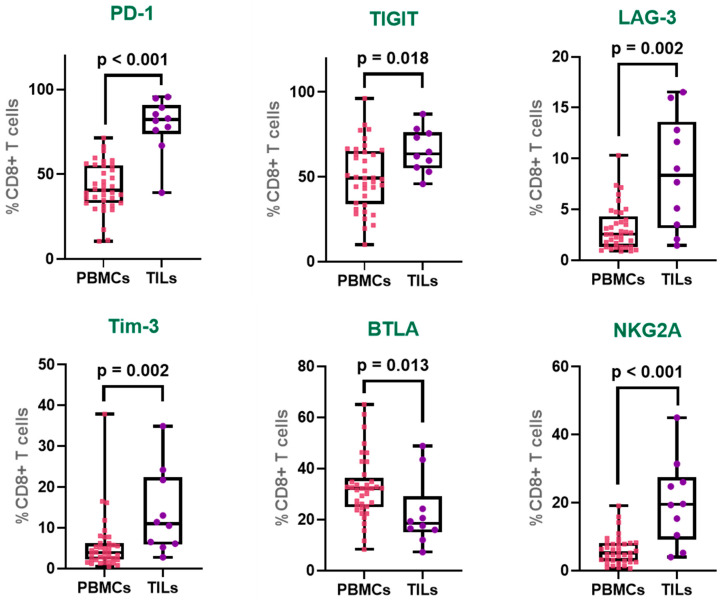
Immune checkpoint expression profiles in CD8^+^ T cells from TILs and PBMCs. Expression of PD-1, TIGIT, LAG-3, Tim-3, BTLA, and NKG2A on tumor-infiltrating CD8^+^ T (TILs) and peripheral CD8^+^ T (PBMCs). The box represents the interquartile range (25th to 75th percentiles), with the center line indicating the median score. The whiskers extend to the min and max of the data set; bold numbers indicate significant results.

In contrast, BTLA exhibited a significantly reduced expression in TILs compared to PBMCs (PBMCs: 32.4%; TILs: 22.6%), suggesting a distinct pattern of regulatory receptor modulation within the tumor microenvironment. Remarkably, NKG2A, a non-classical inhibitory receptor more typically associated with NK cells, was substantially increased in tumor-infiltrating CD8^+^ T cells, showing a 3.4-fold rise relative to peripheral blood (PBMCs: 5.9%; TILs: 20.1%) (Figure 3).

Collectively, these findings highlight a pronounced upregulation of multiple immune checkpoints in tumor-infiltrating CD8^+^ T cells, consistent with an exhausted phenotype shaped by the chronic antigen exposure and immunosuppressive milieu of the tumor microenvironment.

### 3.5. Co-Expression of Immune Checkpoints in CD8^+^ T Cells

**Figure 4:**
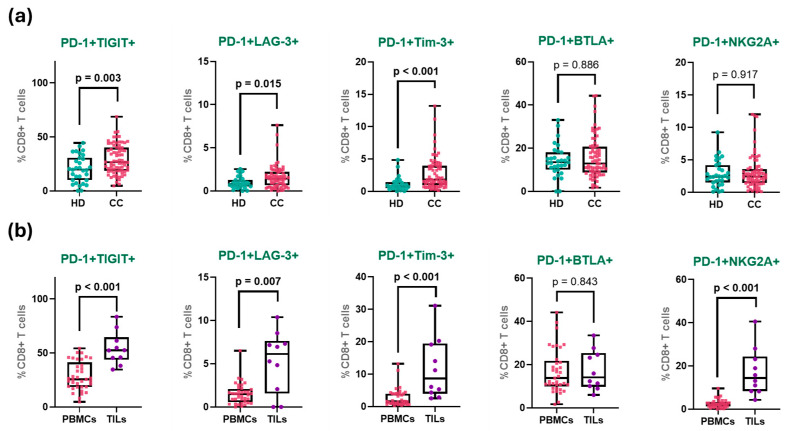
Co-expression of immune checkpoints in CD8^+^ T cell groups. (**a**) Percentage of PD-1 co-expression with inhibitory receptors in peripheral CD8^+^ T cells from HD and all patients with CC. (**b**) Percentage of co-expression in peripheral and tumor-infiltrating CD8^+^ T cells CD8^+^ T cells from treatment-naive patients. The box represents the interquartile range (25th to 75th percentiles), with the center line indicating the median score. The whiskers extend to the min and max of the data set; bold numbers indicate significant results.

To further characterize the exhaustion phenotype of CD8^+^ T cells in cervical cancer, we analyzed the co-expression of inhibitory receptors within the PD-1^+^ CD8^+^ T cell population (Table 2). The simultaneous expression of multiple immune checkpoints is a hallmark of functional exhaustion. Given that PD-1 was the most prominently expressed receptor in our prior analyses, it was selected as the reference marker. We assessed its co-expression with TIGIT, Tim-3, LAG-3, BTLA, and NKG2A across four groups: treatment-naïve CC patients and total CC patients (Figure 4a), and peripheral CD8^+^ T cells from peripheral treatment-naive PBMCs versus tumor-infiltrating CD8^+^ T cells from biopsies of treatment-naive tumors (Figure 4b).

The frequency of PD-1^+^TIGIT^+^ co-expression exhibited a clear gradient across the study groups. In healthy donors, this population was detected at relatively low levels, consistent with a predominantly non-exhausted, resting T cell profile. A moderate increase was observed in treatment-naïve patients, with a slightly higher frequency in treated patients, suggesting a peripheral shift toward an exhausted phenotype, potentially driven by chronic antigen exposure or therapy-induced stress. However, no significant change in PD-1^+^TIGIT^+^ (nor any of the other co-expression populations) was observed, even when matched pairs of samples from the 11 patients that had samples both before and after treatment were analyzed (Supplementary Figure S4).

Notably, the highest frequency of PD-1^+^TIGIT^+^ cells was observed in TILs, where over half of the CD8^+^ T cells co-expressed both receptors (HD: 20.3%; treatment-naïve: 28.9%; treated: 30.2%; TILs: 54.3%). When analyzing matched pairs of TILs and PBMCs from the same patients (only five samples), PD-1^+^TIGIT^+^ and PD-1^+^NKG2A^+^ were the only significantly increased (Supplementary Figure S5).

A similar distribution pattern was observed for PD-1^+^Tim-3^+^ CD8^+^ T cells. This population was virtually absent in healthy donors, reflecting the quiescent state of peripheral CD8^+^ T cells in the absence of chronic antigenic stimulation. In both treatment-naïve and treated CC patients, a modest increase was noted. In stark contrast, tumor-infiltrating lymphocytes exhibited a markedly higher frequency of PD-1^+^Tim-3^+^ cells, representing more than a 4-fold increase compared to peripheral levels (HD: 1.1%; treatment-naïve: 2.8%; treated: 2.7%; TILs: 11.7%).

The co-expression of PD-1 and LAG-3 showed a similar pattern: rare in healthy donors and low in both treatment-naïve and treated patients, but significantly enriched in TILs. Although the absolute frequencies were lower than those of the PD-1^+^TIGIT^+^ and PD-1^+^Tim-3^+^ populations, the relative increase within the tumor remained substantial (HD: 1.0%; treatment-naïve: 1.5%; treated: 1.7%; TILs: 5.3%).

In contrast, the PD-1^+^BTLA^+^ CD8^+^ T cell population displayed a more uniform distribution across groups. While there was a slight upward trend from healthy donors to tumor-infiltrating cells, none of the differences reached statistical significance (HD: 14.2%; treatment-naïve: 16.9%; treated: 14.5%; TILs: 17.3%).

Finally, the most pronounced differential expression was observed in the PD-1^+^NKG2A^+^ CD8^+^ T cell subset. In all peripheral groups, including both patient cohorts and healthy donors, the frequency of this population remained low and relatively stable. However, within TILs, there was a dramatic increase, with frequencies more than six-fold higher than the treatment-naive peripheral group (HD: 2.8%; treatment-naïve: 2.6%; treated: 3.5%; TILs: 17.2%).

### 3.6. Expression of Immune Checkpoints in Cervical Tumors

To characterize the local immune landscape in cervical cancer, we performed immunohistochemical analysis on nine tumor biopsies, assessing the expression and spatial distribution of key immune markers (Supplementary Figure S6): CD8, PD-1, PD-L1, and TIGIT. The median patient age was 34 years (range: 25–72), with 44% with early-stage tumors (IA1-IB2) and 56% presenting with locally advanced disease (IB3-IIIC2).

**Figure 5:**
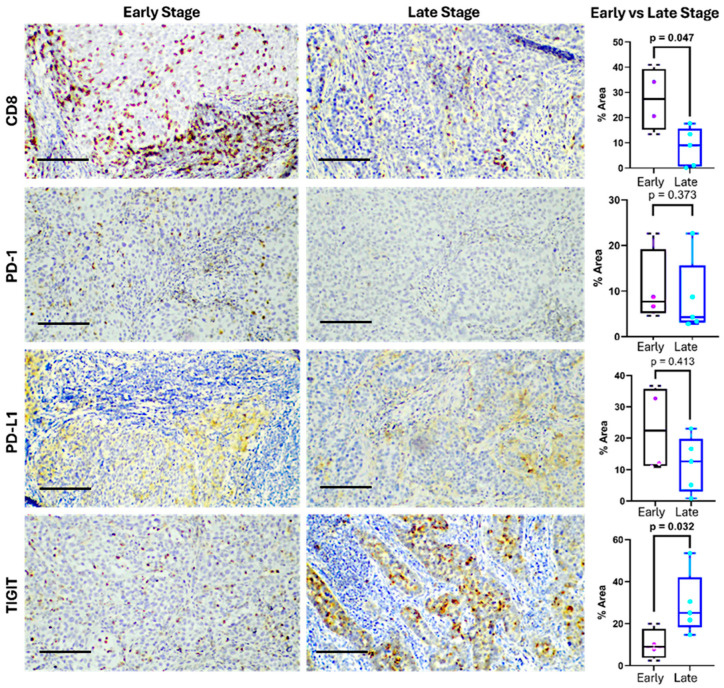
Expression of immune checkpoints in cervical cancer at early and late stages. Representative immunohistochemistry images (left) showing CD8, PD-1, PD-L1, and TIGIT expression in tumor tissues from early- and late-stage cervical cancer patients. The bar indicates 100 µm. Differences in the positive staining area are shown on the right. The box represents the interquartile range (25th to 75th percentiles), with the center line indicating the median score. The whiskers extend to the min and max of the data set; bold numbers indicate significant results.

Notably, a marked difference between early and advanced biopsies was observed. Early-stage tumors were characterized by an increased CD8^+^ T cell infiltration (27.29% early, 8.27% advanced), and advanced tumors showed higher expression of TIGIT (10.09% early, 29.13% advanced) (Figure 5).

CD8^+^ T cells displayed strong membranous and cytoplasmic staining, primarily within the stromal compartment. Their expression ranged from 0.15% to 40.95%, with a median of 16.21%. CD8^+^ infiltration was observed in eight of nine samples, indicating a frequent, although heterogeneous and lower in advanced stages, presence of cytotoxic lymphocytes.

PD-1 expression was confined to lymphocytic infiltrates and showed a median of 8.55% (range: 2.85–24.22%), suggesting the presence of antigen-experienced or exhausted T cells. PD-L1 was detected in both tumor and immune cells, with a median expression of 15.68% (range: 0.85–36.37%). Staining appeared cytoplasmic, occasionally paranuclear, and was diffusely distributed in tumor areas, consistent with tumor-intrinsic immune evasion via the PD-1/PD-L1 axis. Eight of the nine samples expressed PD-L1 over 1%.

TIGIT showed the highest expression (median: 18.68%; range: 2.43–53.55%), with granular cytoplasmic staining in both lymphocytes and tumor cells. In most samples, TIGIT levels exceeded those of PD-1, highlighting its potential dominance as an inhibitory pathway in this context, especially in locally advanced tumors. Overall, eight of the nine tumors co-expressed CD8, PD-1, PD-L1, and TIGIT, revealing a microenvironment rich in T cells yet shaped by strong immunosuppressive signals (Figure 5).

## 4. Discussion

T cell exhaustion is a major contributor to immune dysfunction in cervical cancer, driven by chronic antigen exposure within the tumor microenvironment. CD8^+^ T cells progressively lose cytotoxicity and cytokine production, marked by an upregulation of inhibitory receptors [17]. In cervical cancer, tumor cells exploit this mechanism by overexpressing checkpoint receptors, promoting T cell dysfunction. While PD-1, PD-L1, and CTLA-4 are well-characterized, emerging checkpoints like LAG-3, Tim-3, and TIGIT also play key roles. These pathways are now central targets of immune checkpoint inhibitors aimed at restoring T cell function and enhancing antitumor responses [18].

In our study, peripheral CD8^+^ T cells from cervical cancer patients showed elevated PD-1 expression, a finding also reported in other cancers [19]. High PD-1 levels in CD8^+^ T cells have been linked to poor survival and may serve as a prognostic marker in malignancy [20]. Notably, in our study, PD-1 expression showed a tendency to increase after standard treatment, possibly due to the radiation. These findings suggest that PD-1 expression dynamics could inform treatment strategies, supporting the concomitant use of PD-1 inhibitors with chemoradiotherapy [21].

We also observed markedly elevated PD-1 on tumor-infiltrating CD8^+^ T cells. Intratumoral PD-1 has been found to correlate with cytokine dysfunction and poor T cell fitness [22,23]. In various cancers, PD-1^+^ TILs are associated with tumor progression and poor prognosis [24]. Specifically in cervical cancer, high PD-1 expression on TILs is associated with early recurrence and poor overall survival [25].

Although blockade of the PD-1/PD-L1 axis has emerged as a prominent immunotherapeutic strategy against invasive cancers, its efficacy is often limited by the presence of terminally exhausted CD8^+^ tumor-infiltrating T cells [26]. These cells have been shown to resist PD-1 blockade, and importantly, to promote the generation and maintenance of aggressive cancer cells [27]. The abundance of this terminally exhausted CD8^+^ T cell population has been identified as a key factor contributing to resistance to immune checkpoint therapy [28].

In addition to PD-1 overexpression, we observed a marked increase in TIGIT expression on CD8^+^ T cells, regardless of treatment status. TIGIT^+^ CD8^+^ T cells exhibit reduced levels of effector molecules such as IFN-γ, perforin, and granzyme B, indicating functional impairment and supporting TIGIT’s role in promoting T cell exhaustion within the tumor microenvironment [29]. Our results show an increased expression of TIGIT in treatment naïve and treated patients with CC compared with HD. Targeting TIGIT could thus be a promising immunotherapeutic strategy. In particular, a dual blockade of TIGIT and PD-1 has been shown to synergistically restore CD8^+^ T cell proliferation, cytokine production, and cytotoxic activity, enhancing antitumor immunity more effectively than either blockade alone [30].

In the same way, we observe an increased LAG-3 expression in peripheral CD8^+^ T cells of patients with CC compared with HD, elevated levels were detected within the tumor microenvironment. Perhaps more importantly, LAG-3 was the only receptor to markedly increase (approximately two-fold, but this result was not significant due to a lack of normality in the data from this test) after standard treatment. LAG-3 is a key immune co-inhibitory receptor alongside PD-1 and TIGIT, reported in various cancers [31]. Its expression on TILs may serve as a prognostic biomarker, particularly for identifying patients likely to benefit from immunotherapy. [32].

Functionally, LAG-3 engagement inhibits T cell activation and proliferation, facilitating tumor immune evasion. Importantly, high frequencies of PD-1/PD-L1^+^ tumors in non-small-cell lung cancer and breast cancer often show co-infiltration with LAG-3^+^ TILs. This supports combined immune checkpoint blockade targeting both PD-1 and LAG-3 pathways, which is under active clinical investigation to improve antitumor immunity and patient outcomes [33,34].

We observed increased Tim-3 expression on peripheral CD8^+^ T cells, with particularly high levels within the tumor microenvironment, where Tim-3 plays a crucial role in immune regulation. Similar findings have been reported in ovarian cancer, where elevated Tim-3 on peripheral CD8^+^ T cells correlates with advanced disease and may promote tumor progression by driving T cell exhaustion and impairing antitumor immunity [35]. In pancreatic cancer, high Tim-3 expression is associated with increased invasion, metastasis, and recurrence, indicating its role in tumor aggressiveness [36].

Additionally, Tim-3 contributes to gastric cancer pathogenesis, serving both as a marker of disease progression and an independent prognostic factor. Together, these studies highlight Tim-3′s key role in mediating immune exhaustion and facilitating tumor progression across multiple cancers, underscoring its promise as a target for immunotherapy [37]. Therapeutic blockade of Tim-3 with monoclonal antibodies has shown potential to restore CD8^+^ T cell function and is actively being investigated, particularly in combination with anti-PD-1 therapies [38].

Regarding BTLA, we found no significant differences in its expression on peripheral CD8^+^ T cells from cervical cancer patients, though a modest decrease was observed within the tumor microenvironment. Elevated BTLA expression has been reported in PBMCs from acute myeloid leukemia patients compared to healthy controls [39]. In other tumors, BTLA is overexpressed in advanced stages and lymphatic invasion, with higher levels linked to shorter relapse-free survival [40]. Similarly, increased BTLA and its ligand HVEM correlate with tumor progression and poor prognosis [41]. Importantly, antagonistic antibodies targeting BTLA can enhance CD8^+^ T cell function and, when combined with other checkpoint inhibitors like PD-1 or Tim-3, exhibit synergistic effects, highlighting BTLA as a promising target for immunotherapy [42].

Finally, although we observed no significant difference in NKG2A expression on peripheral CD8^+^ T cells between healthy donors and cervical cancer patients, a marked increase was detected in TILs. This finding aligns with reports of elevated NKG2A expression on tumor-infiltrating CD8^+^ T cells [43]. Moreover, heavy infiltration of NKG2A^+^ CD8^+^ T cells has been linked to poorer prognosis and reduced immunotherapy response in renal cell carcinoma [44]. Consequently, NKG2A represents a promising therapeutic target, with ongoing studies investigating its blockade to enhance antitumor CD8^+^ T cell responses [45].

The increased co-expression of immune checkpoints is a hallmark of the exhausted CD8^+^ T cell phenotype, with PD-1 serving as a key indicator of dysfunction. Here, we observed elevated co-expression of PD-1 with TIGIT, TIM-3, LAG-3, and NKG2A, which was particularly pronounced in tumor-infiltrating CD8^+^ T cells compared to peripheral blood. These findings align with our previous reports of immune checkpoint overexpression in cervical cancer patient T cells, both in peripheral blood [46] and within the tumor, and highlight the importance of the tumor microenvironment versus PBMCs [47]. The simultaneous upregulation of multiple inhibitory receptors underscores the complex regulatory networks driving T cell exhaustion in the tumor microenvironment and supports the rationale for combination checkpoint blockade therapies to restore antitumor immunity.

Similar co-expression patterns have been described in other malignancies. For instance, in non-small-cell lung cancer, PD-1^+^TIGIT^+^ CD8^+^ T cells are enriched in tumors versus peripheral blood, correlating with impaired function [29]. In acute myeloid leukemia, these exhaustion markers are linked to poor clinical outcomes, reflecting their negative impact on antitumor immunity [48]. Furthermore, tissue-resident memory T cells co-expressing PD-1 and TIGIT in endometrial cancer show reduced cytotoxicity and tumor targeting; yet, they paradoxically maintain enhanced proliferation, potentially contributing to their persistence within the tumor. This duality highlights the complex roles of PD-1 and TIGIT in modulating immune responses in cancer [49].

In the same way, other combinations of immune checkpoints have emerged as important markers defining exhausted CD8^+^ T cells. In ovarian cancer, PD-1^+^Tim-3^+^ CD8^+^ T cells exhibit classic features of functional exhaustion and correlate with poor clinical outcomes [50]. In follicular lymphoma, LAG-3 expression distinguishes functionally exhausted intratumoral PD-1^+^ T cells from PD-1^+^LAG-3^−^ cells, which remain activated and immunologically competent [51]. The co-expression of PD-1 and LAG-3 in these cells correlates with impaired cytotoxicity and reduced cytokine production. Additionally, tumor-specific CD8^+^ T lymphocytes often co-express PD-1 and BTLA, with BTLA^+^PD-1^+^ cells displaying greater dysfunction, including reduced IFN-γ production compared to BTLA^−^PD-1^+^ cells [52].

Additionally, analysis of tumor biopsies using advanced digital pathology tools revealed a high expression not only of inhibitory receptors such as PD-1 and TIGIT, but also of PD-L1, indicating a strongly immunosuppressive microenvironment. Elevated PD-L1 levels detected by immunohistochemistry are generally correlated with improved clinical responses to immune checkpoint blockade therapies [53]. In the cohort of early- and advanced-stage patients utilized for the immunohistochemistry section of this study, PD-L1 was not increased in advanced tumors, but it must be highlighted that these were treatment-naïve samples; standard treatment has been reported to increase PD-L1 expression, and thus biopsies post-treatment would be expected to show a different microenvironment [21]. Notably, we observed increased TIGIT in advanced stages, as well as decreased CD8^+^ T cells. Interestingly, CD155, the ligand for TIGIT, was found to be highly expressed in cervical cancer tissues and negatively correlated with infiltrating CD8^+^ T cell percentages [54]. While the literature shows that checkpoint expression is often heterogeneous with respect to stage, we posit that irrespective of early- or advanced-stage, increased checkpoint expression leads to increasingly dysfunctional T cells, and that these cells, in addition to being cytotoxically impaired, might not traffic as efficiently to tumor sites, and if they do manage to infiltrate tumors, they are more likely to be further stimulated to express multiple checkpoint receptors, and more likely to differentiate to a terminally exhausted phenotype, which are shorter lived that normal cytotoxic T cells [55]. These results underscore the value of using powerful digital pathology methods to quantify immune checkpoint molecules in biopsies, highlighting the critical role of the tumor microenvironment in driving T cell exhaustion and reinforcing these pathways as promising targets for novel immunotherapies.

To summarize the above discussion, chemotherapy and radiotherapy, while essential components of cervical cancer treatment, have been shown to induce immune modulation that may contribute to T cell dysfunction. Notably, several studies have reported an increase in PD-1 expression on peripheral and tumor-infiltrating CD8^+^ T cells following chemoradiotherapy in cervical cancer patients [21], suggesting that treatment-induced stress and chronic antigen exposure promote T cell exhaustion [56].

This upregulation of inhibitory receptors, including LAG-3, TIGIT, and Tim-3, in addition to, and sometimes in combination with, PD-1 [21,34,57,58,59,60], can impair effective anti-tumor immune responses and may partially explain the reduced CD8^+^ T cell infiltration observed in biopsies from patients with advanced disease.

Furthermore, persistent infection with high-risk human papillomavirus (HPV), the etiologic agent of cervical cancer, provides a continuous antigenic stimulus that drives the expression of multiple exhaustion markers on CD8^+^ T lymphocytes [61,62]. Thus, it is very likely that the immunosuppressive microenvironment shaped by chronic HPV infection and treatment-associated immune stress contributes to the functional impairment and numerical decline of cytotoxic T cells in the tumor.

These insights are clinically relevant as immune checkpoint inhibitors, particularly the PD-1/PD-L1 blockade, have become the standard of care as a first-line or combined treatment for persistent, recurrent, or metastatic cervical cancer patients with a combined positive score equal or greater than 1 (and are showing positive results with high-risk locally advanced patients), highlighting the therapeutic potential of combined therapies reversing T cell exhaustion to improve anti-tumor immunity and patient outcomes [63,64].

Collectively, these findings underscore the complex interplay between therapeutic interventions, viral antigen presence, and immune checkpoint regulation in shaping the CD8^+^ T cell landscape in cervical cancer.

While multivariate statistical models can be valuable for identifying predictive or prognostic biomarker combinations, the design and scope of the present exploratory study were not optimized for such analyses. Our primary aim was to characterize the expression and co-expression of established and emerging immune checkpoint molecules in cervical cancer, rather than to construct predictive models of clinical or treatment outcomes. Multivariate modeling to distinguish tumor versus peripheral samples was not appropriate because these represent biologically distinct compartments, and group identity is determined a priori by tissue origin. Likewise, modeling treatment status as a dependent variable was not meaningful, as no checkpoint marker showed a significant treatment-related difference in univariate comparisons, indicating that there was insufficient signal for regression modeling. Future studies incorporating longitudinal clinical follow-up, treatment response, and survival data will be essential to enable robust multivariate analyses aimed at defining prognostic or predictive immune signatures in cervical cancer.

One principal weakness of this study is the lack of correlation of increased immune checkpoint expression, particularly PD-1 and TIGIT, with treatment. One simple solution would be to recruit more patients in both treated and treatment-naïve arms of the study, and to recruit more matched tumor biopsies. Related to this is the less-than-ideal experimental design inherent when one is working with a heterogeneity of treated patients in which “treated” patients may have received different therapies or treatment durations, and in which treated patients had different clinical status entering the protocol, introducing variability that may confound interpretation. We do not have treatment duration information for this study, and the 36 patients with therapy were divided between the 5 patients with surgery (early-stage) 4 with chemotherapy only (metastatic) and 27 patients who received radiotherapy with or without chemotherapy; most underwent concurrent chemoradiotherapy, consistent with current treatment standards for locally advanced cervical cancer (81% of our cohort). However, given the importance of the tumor microenvironment that this study has found, it would be more interesting to obtain biopsies from patients post standard treatment (with a better defined and limited treatment cohort), and analyze both the TILs and in situ expression of checkpoint receptors and ligands. This would be important particularly in the case of PD-1 and PD-L1, which have already been reported by some groups, but doubly so in the case of TIGIT, TIM-3, LAG-3, and NKG2A (and their respective ligands).

## 5. Conclusions

This study highlights the importance of examining the tumor microenvironment of cervical cancer directly. Both in terms of the analysis of TILs and of immunohistochemistry, the crucial role of multiple immune checkpoints, including PD-1, TIGIT, TIM-3, LAG-3, BTLA, and NKG2A, in driving CD8^+^ T cell exhaustion in cervical cancer becomes clearer when focusing on the transformed tissue and infiltrating cells. The co-expression of these inhibitory receptors, especially within tumor-infiltrating lymphocytes, reflects the complex immunosuppressive environment established by the tumor. Notably, systemic treatments like chemoradiotherapy can further modulate these pathways, as shown by increased LAG-3 expression, and the trend to increased PD-1, post-therapy, potentially sustaining T cell dysfunction and immune evasion. The persistence of T cells co-expressing multiple checkpoints highlights the limitations of single-agent therapies and supports the need for combination immunotherapies targeting several inhibitory pathways simultaneously. As clinical trials advance, identifying exhaustion signatures in cervical cancer will be crucial to tailor therapies and overcome the strong immunosuppressive tumor microenvironment, ultimately enhancing antitumor immune responses and patient outcomes.

## Figures and Tables

**Table 1:** Clinical characteristics.

Variable	Healthy Donors *n* = 31	Treatment-Naïve *n* = 37	Treated *n* = 36
Age (range)	40 (25–72)	45 (25–85)	43 (25–66)
**Histological subtype**			
Squamous cell carcinoma	-	33 (89.2%)	24 (66.7%)
Adenocarcinoma	-	4 (10.8%)	12 (33.3%)
**Clinical FIGO staging**			
Early-stage (IA1-IB1)	-	5 (13.5%)	4 (11.1%)
Locally advanced (IB2-IVA)	-	32 (86.5%)	31 (86.1%)
Metastatic (IVB)	-	0 (0%)	1 (2.8%)
**Primary treatment**			
Surgery only	-	-	5 (13.9%)
Radiotherapy with or without chemotherapy	-	-	27 (75%)
Chemotherapy only	-	-	4 (11.1%)

**Table 2:** Immune checkpoint expression in patients with cervical cancer.

Immune Checkpoint Expression in Peripheral CD8^+^ T Cells							
	HD			CC			*p*
	Median	25%	75%	Median	25%	75%	
PD-1	35.47	25.03	52.02	44.5	36.11	55.94	**0.005**
TIGIT	38.6	22.39	50.69	53.05	36.47	64.37	**<0.001**
LAG-3	1.77	1.28	2.73	2.94	1.645	4.815	**0.002**
Tim-3	1.61	1.18	2.3	3.56	2.285	6.21	**<0.001**
BTLA	32.64	24.91	38.54	32.15	24.23	37.19	0.709
NKG2A	5.5	3.53	8.18	5.58	3.315	8.16	0.667
Immune checkpoint expression in peripheral CD8^+^ T cells before and after treatment							
	HD			CC			*p*
	Median	25%	75%	Median	25%	75%	
PD-1	40.57	33.03	55.36	50.58	40.34	57.98	0.064
TIGIT	49.24	34.01	65.21	56.51	38.83	63.68	0.498
LAG-3	2.56	1.315	4.325	3.44	2.185	6.035	0.095
Tim-3	3.95	2.17	6.21	3.27	2.628	6.458	0.971
BTLA	32.15	24.79	36.24	31.7	21.95	38.08	>0.999
NKG2A	5.15	2.705	8.135	5.625	3.808	8.473	0.474
Immune checkpoint expression profiles in CD8^+^ T cells from TILs and PBMCs							
	PBMCs			TILs			*p*
	Median	25%	75%	Median	25%	75%	
PD-1	40.57	33.03	55.36	82.3	73.76	90.81	**<0.001**
TIGIT	49.24	34.01	65.21	63.48	55.01	76.3	**0.018**
LAG-3	2.56	1.315	4.325	8.34	3.15	13.6	**0.002**
Tim-3	3.95	2.17	6.21	10.96	5.895	22.36	**0.002**
BTLA	32.15	24.79	36.24	18.44	14.96	29.02	0.0133
NKG2A	5.15	2.705	8.135	19.46	9.128	27.41	**<0.001**
Co-expression of inhibitory receptors in peripheral CD8^+^ T cells from HD and CC							
	HD			CC			*p*
	Median	25%	75%	Median	25%	75%	
PD-1^+^TIGIT^+^	19.72	10	30.76	26.65	18.58	40.26	**0.003**
PD-1^+^Tim-3^+^	0.84	0.52	1.41	1.82	0.99	3.97	**<0.001**
PD-1^+^LAG-3^+^	0.82	0.54	1.27	1.42	0.65	2.205	**0.015**
PD-1^+^BTLA^+^	13.47	10.04	18.05	12.9	8.76	20.75	0.886
PD-1^+^NKG2A^+^	2.41	1.51	4.17	2.44	1.405	3.54	0.917
Co-expression of inhibitory receptors in treatment-naive peripheral and tumor-infiltrating CD8^+^ T cells							
	PBMCs			TILs			*p*
	Median	25%	75%	Median	25%	75%	
PD-1^+^TIGIT^+^	25.66	18.58	41.48	52.58	43.72	64.53	**<0.001**
PD-1^+^Tim-3^+^	1.66	0.87	3.99	8.65	3.975	19.44	**<0.001**
PD-1^+^LAG-3^+^	1.47	0.57	2.1	6.12	1.553	7.625	0.077
PD-1^+^BTLA^+^	13.75	9.9	21.74	14.12	9.785	25.34	0.843
PD-1^+^NKG2A^+^	2.33	1.26	3.265	14.45	8.363	24.41	**<0.001**

Bold indicates significant results.

## Data Availability

The data presented in this study, after anonymization of patient identifiers, due to patient privacy and institutional regulations, are available on request from the corresponding authors.

## Supplementary Materials

The following supporting information can be downloaded at https://www.mdpi.com/article/10.3390/cancers17223627/s1, Figure S1: Gating strategy; Figure S2: MFI of immune checkpoint expression in peripheral CD8^+^ T cells; Figure S3: Differential expression of immune checkpoints on CD8^+^ T cells by histological subtype; Figure S4: Co-expression of immunological receptors in CD8 TILs versus PBMCs from the same patients: Figure S5: Co-expression of inhibitory immunological receptors in PBMCs in the follow-up of 11 patients with cervical cancer matched pre- and post-treatment. Figure S6: Characteristics and results of Immunohistochemistry in patients with cervical cancer.
