# Supplementary material for: T Cell Exhaustion in the Cervical Cancer Tumor Microenvironment: PD-1 Overexpression and Co-Expression with TIGIT, Tim-3, LAG-3, and NKG2A
Source: Cancers (Basel). 2025 Nov 11;17(22):3627. doi: 10.3390/cancers17223627 (PMC12651438; doi:10.3390/cancers17223627)
Supplement: Supplementary file 1 [file cancers-17-03627-s001.zip › cancers-3920351-supplementary.pdf]

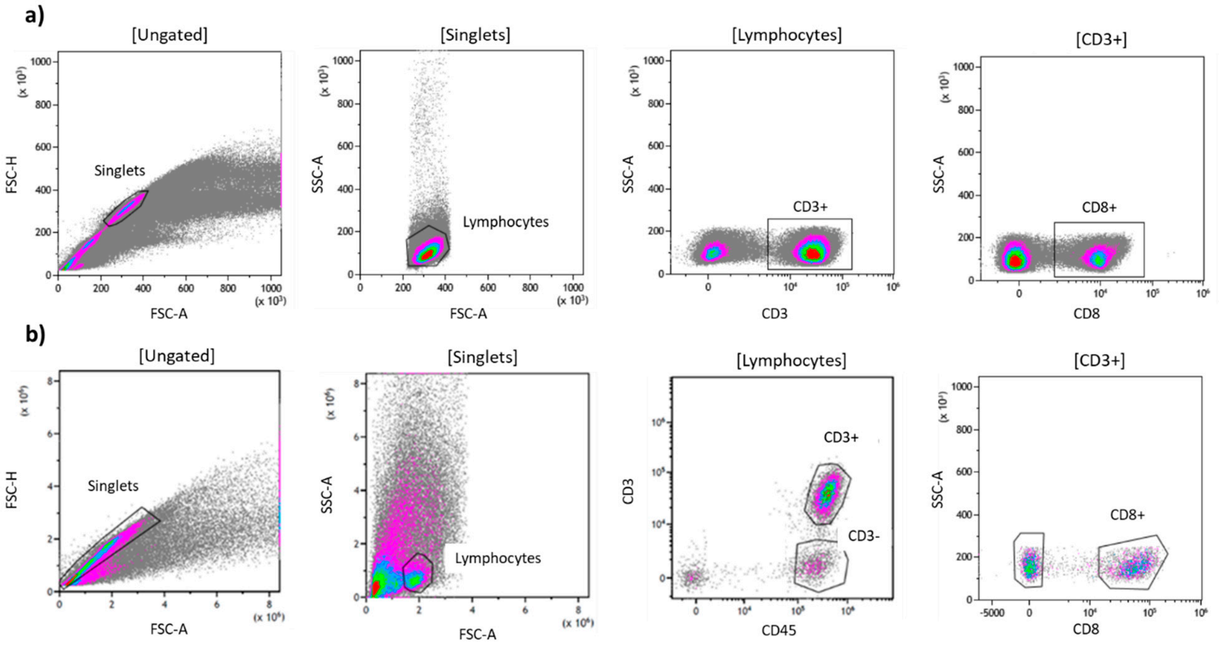

Supplementary Figure S1. Gating strategy. a) Gating strategy for peripheral CD8$^+$ T cells. b) Gating strategy for tumor-infiltrating CD8$^+$ T cells.

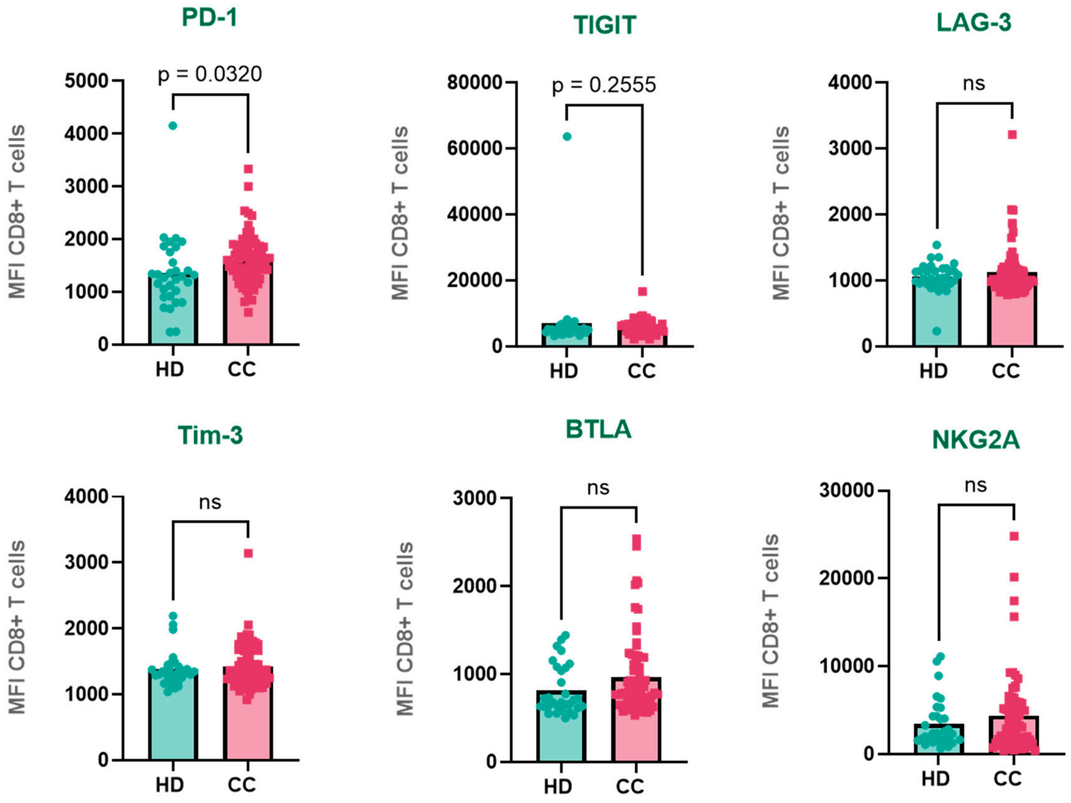

Supplementary Figure S2. MFI of immune checkpoint expression in peripheral $CD8^+$ T cells. MFI values of PD-1, TIGIT, LAG-3, Tim-3, BTLA, and NKG2A on $CD8^+$ T cells from cervical cancer patients and healthy donors; ns = non-significant.

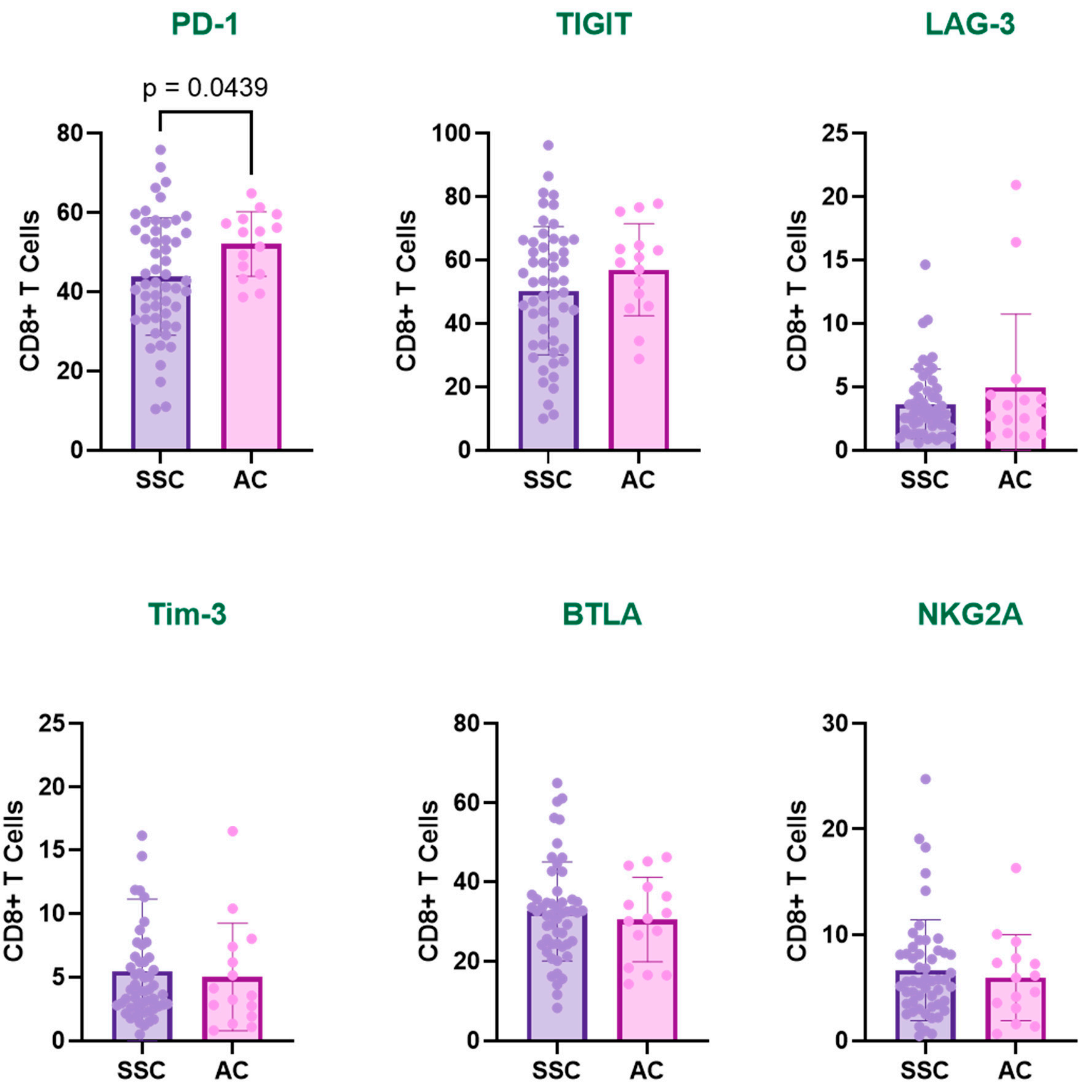

Supplementary Figure S3: Differential expression of immune checkpoints on $CD8^+$ T cells by histological subtype**.** Comparison of PD-1 levels in peripheral $CD8^+$ T cells from adenocarcinoma and squamous cell carcinoma.

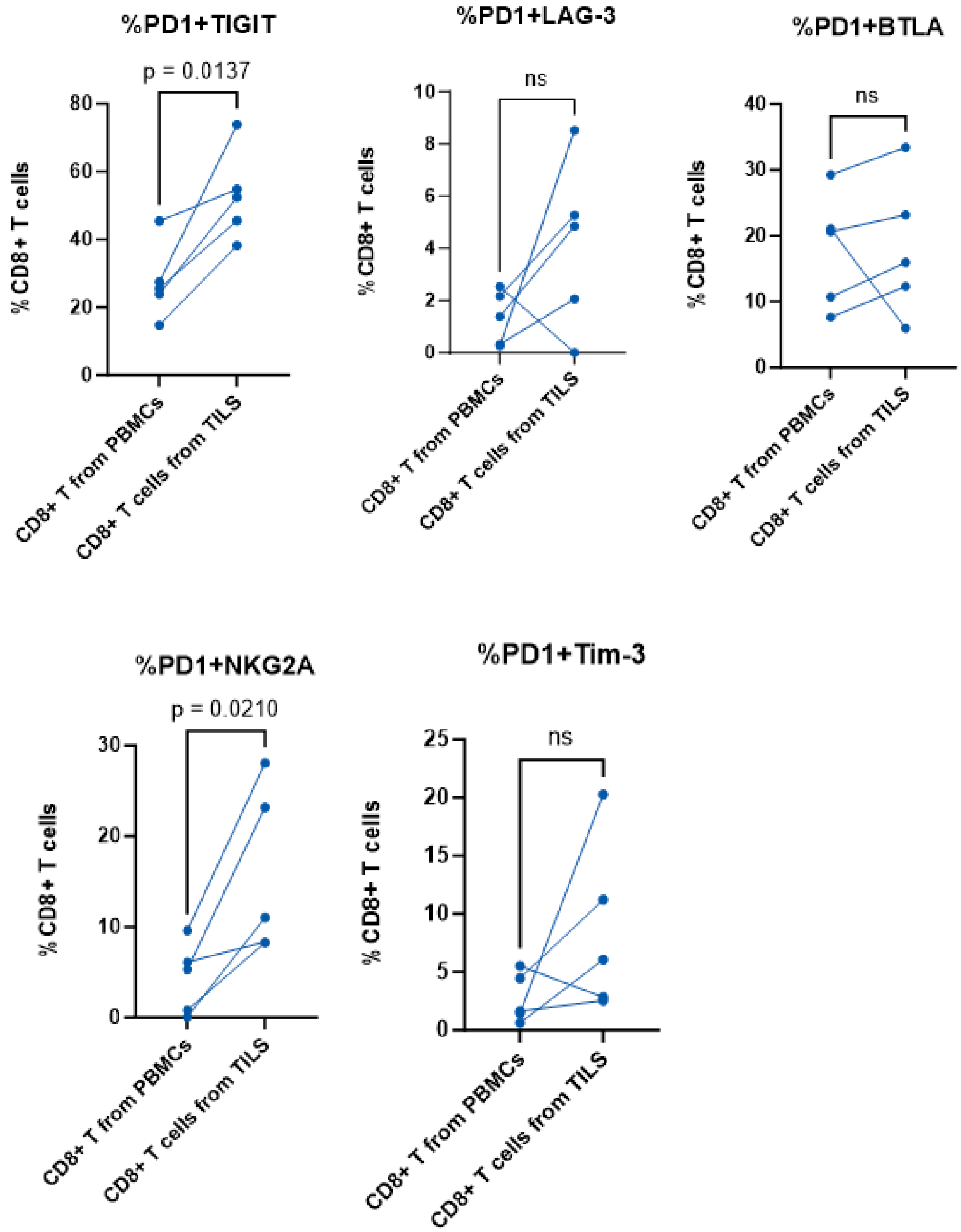

Supplementary Figure S4. Co-expression of inhibitory immunological receptors in CD8^{+} TILs versus CD8^{+} PBMCs from the same patients, 5 patients with cervical cancer; ns = non-significant.

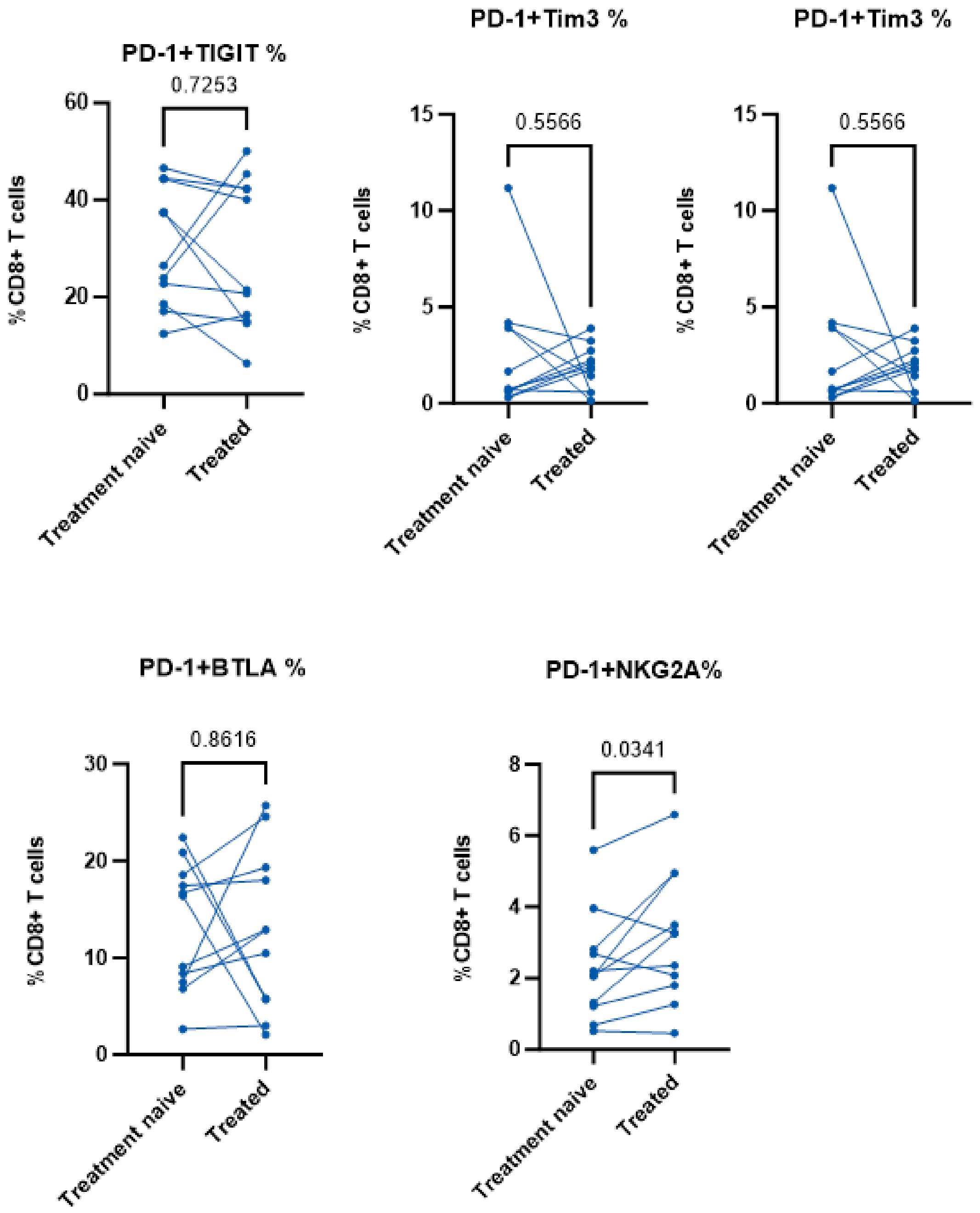

Supplementary Figure S5. Co-expression of inhibitory immunological receptors in PBMCs in the follow-up of 11 patients with cervical cancer, matched pre- and post- treatment.

Early Stage					
**Age (years)**	**Stage (FIGO)**	**CD8 x̄=27.29%**	**PD-1 x̄=10.67%**	**PD-L1 x̄=23.08%**	**TIGIT x̄=10.09%**
25	IA2	34.18%	8.73%	10.94%	7.81%
29	IB	13.39%	22.66%	32.68%	10.18%
27	IB2	40.95%	4.60%	11.96%	19.94%
33	IB2	20.62%	6.67%	36.72%	2.43%

Late Stage					
**Age (years)**	**Stage (FIGO)**	**CD8 x̄=8.27%**	**PD-1 x̄=8.38%**	**PD-L1 x̄=11.61%**	**TIGIT x̄=29.13%**
33	IIA	0.15%	8.73%	0.85%	25.14%
72	IIIA	1.03%	22.66%	16.54%	53.51%
36	IIIC	9.06%	4.31%	12.57%	30.54%
38	IIIB	13.42%	3.36%	23.00%	21.73%
42	IIIC2	17.68%	2.85%	5.10%	14.74%

Supplementary Figure S6. Characteristics and results of Immunohistochemistry in patients with cervical cancer.
